# Vitamin E Delivery Systems Increase Resistance to Oxidative Stress in Red Deer Sperm Cells: Hydrogel and Nanoemulsion Carriers

**DOI:** 10.3390/antiox10111780

**Published:** 2021-11-06

**Authors:** Alejandro Jurado-Campos, Pedro Javier Soria-Meneses, Francisca Sánchez-Rubio, Enrique Niza, Iván Bravo, Carlos Alonso-Moreno, María Arenas-Moreira, Olga García-Álvarez, Ana Josefa Soler, José Julián Garde, María del Rocío Fernández-Santos

**Affiliations:** 1 SaBio IREC (CSIC—UCLM—JCCM), Campus Universitario, s/n, 02071 Albacete, Spain; alejandro.jurado@uclm.es (A.J.-C.); PedroJavier.Soria@uclm.es (P.J.S.-M.); paki_sr@hotmail.com (F.S.-R.); mariaarenasmoreira@gmail.com (M.A.-M.); olga.garcia@uclm.es (O.G.-Á); AnaJosefa.Soler@uclm.es (A.J.S.); Julian.Garde@uclm.es (J.J.G.); 2 Servicio de Farmacia Hospitalaria, Complejo Hospitalario Universitario de Albacete, GAI, 02071 Albacete, Spain; 3 Centro Regional de Investigaciones Biomédicas, Unidad Nano-CRIB, 02071 Albacete, Spain; Enrique.Niza@uclm.es (E.N.); ivan.bravo@uclm.es (I.B.); Carlos.AMoreno@uclm.es (C.A.-M.); 4 Facultad de Farmacia, Universidad de Castilla la Mancha, 02071 Albacete, Spain

**Keywords:** hydrogel, nanoemulsions, vitamin E, sperm oxidative stress, antioxidant

## Abstract

Oxidative stress has become a major concern in the field of spermatology, and one of the possible solutions to this acute problem would be the use of antioxidant protection; however, more studies are required in this field, as highly contradictory results regarding the addition of antioxidants have been obtained. Vitamin E is a powerful biological antioxidant, but its low stability and high hydrophobicity limit its application in spermatology, making the use of organic solvents necessary, which renders spermatozoa practically motionless. Keeping this in mind, we propose the use of hydrogels (HVEs) and nanoemulsions (NVEs), alone or in combination, as carriers for the controlled release of vitamin E, thus, improving its solubility and stability and preventing oxidative stress in sperm cells. Cryopreserved sperm from six stags was thawed and extended to 30 × 10^6^ sperm/mL in Bovine Gamete Medium (BGM). Once aliquoted, the samples were incubated as follows: control, free vitamin E (1 mM), NVEs (9 mM), HVEs (1 mM), and the combination of HVEs and NVEs (H + N), with or without induced oxidative stress (100 µM Fe^2+^/ascorbate). The different treatments were analyzed after 0, 2, 5, and 24 h of incubation at 37 °C. Motility (CASA^®^), viability (YO-PRO-1/IP), mitochondrial membrane potential (Mitotracker Deep Red 633), lipid peroxidation (C_11_ BODIPY 581/591), intracellular reactive oxygen species production (CM-H_2_DCFDA), and DNA status (SCSA^®^) were assessed. Our results show that the deleterious effects of exogenous oxidative stress were prevented by the vitamin E-loaded carriers proposed, while the kinematic sperm parameters (*p* ˂ 0.05) and sperm viability were always preserved. Moreover, the vitamin E formulations maintained and preserved mitochondrial activity, prevented sperm lipid peroxidation, and decreased reactive oxygen species (ROS) production (*p* ˂ 0.05) under oxidative stress conditions. Vitamin E formulations were significantly different as regards the free vitamin E samples (*p* < 0.001), whose sperm kinematic parameters drastically decreased. This is the first time that vitamin E has been formulated as hydrogels. This new formulation could be highly relevant for sperm physiology preservation, signifying an excellent approach against sperm oxidative damage.

## 1. Introduction

Oxidative stress is one of the main concerns in spermatology, and is considered an unbridgeable factor in the field of fertility. However, the balance between reactive oxygen species (ROS) production and antioxidant activity is delicate, and therefore a physiological concentration of ROS is necessary for some biological processes, such as promoting the signal transduction pathways associated with capacitation or the maintenance of the spermatozoa motility. Control over this balance is crucial as regards avoiding sperm damage, and the most widely used means in the field of spermatology is the addition of antioxidants. In this regard, the choice and dosage of antioxidants are critical; an insufficient supplementation may cause a reduction in motility and mitochondrial activity [1,2], an increase in lipid peroxidation, and ROS [2,3,4], and DNA oxidation and fragmentation [5,6], while an excessive supplementation is harmful [7,8,9,10,11].

Many strategies for avoiding the negative effects of oxidative stress, improving sperm quality, and minimizing cytotoxicity have been proposed [12]. In this context, our research group has studied the use of a wide range of antioxidants, such as vitamin E [7,8,9,10,12,13,14,15,16]; catalase and superoxide dismutase [10]; melatonin [8,9]; crocin [8]; dehydroascorbic acid (DHA); TEMPOL, N-acetyl-cysteine (NAC), and rutin [11]; and cinnamtannin B-1 (CNB1) [17]. Among the most relevant results founds, we have proven that antioxidants improve the viability of sperm samples, whether refrigerated [18], cryopreserved [7,10,13], or incubated after thawing [8]. Moreover, spermatozoa viability during sperm separation X and Y through flow cytometry was enhanced by antioxidants [13].

The antioxidative properties of vitamin E is a benchmark in spermatology because it can protect sperm samples by reducing ROS production and lipid peroxidation. Moreover, DNA and acrosome damage are reduced by vitamin E [8]. However, this antioxidant has important limitations, including instability against oxidative processes or pH changes, the possibility of having prooxidant effects at relatively low concentrations, and low solubility. Moreover, the low solubility of vitamin E signifies that it must be administered with organic solvents, such as ethanol or dimethyl sulfoxide, which are toxic to sperm cells [19] and associated with a loss of sperm motility [7,8,9,13,20].

Recently, we have shown that the controlled release of vitamin E carried by nanoemulsions prevents negative effects of oxidative stress while eliminating the use of organic solvents, thus, avoiding the loss of sperm motility [12]. Nanoemulsions are vesicular systems comprising an oily core and a surfactant, which is used to give the formulation stability [21]. Therefore, in this study, we propose the use of other delivery systems such as hydrogels. Hydrogels have a great capacity to embed water or other biological fluids in their three-dimensional network formed of cross-linked monomers [22]. Furthermore, hydrogels can easily encapsulate hydrophilic compounds of different sizes and properties [23]. The usage of hydrogels is currently widespread in nanotechnology, and recent advances in bioengineering and nanotechnology have increased the developments of new therapeutic drug delivery systems [24,25], showing that hydrogels are excellent systems for oral, rectal, ocular, epidermal, and subcutaneous applications [26,27]. The good results obtained after combining hydrogels with others nanocarriers, such as nanoemulsions or nanoparticles, have led us to believe that hydrogels may be perfect candidates for the co-delivery of drugs into spermatozoa. These co-delivery systems have the potential characteristics of a hydrogel (e.g., 3D hydrophilic networks, chemoselectibility, biodegradability, and an extremely high loading efficiency) [27] with a nanoemulsion (e.g., very small and a huge surface area) [28]. Recently, Ridzewski et al. (2020) proved that gelatin spermbots, using a polycarbonate template, have great potential for the development of noninvasive theranostic tools in reproductive biology and medicine for the protection and activation of sperm, which is especially useful when studying sperm migration. Their beneficial features include biocompatibility and biodegradability, as well as pH response, loading stability, and antioxidant protection [29]. Keeping all these results in mind, we propose the use of hydrogels for the controlled release of VE to prevent oxidate stress in spermatology. To the best our knowledge, the use of hydrogels as antioxidant carriers against oxidative stress in spermatology has never been studied. As a proof of concept, we evaluated the effects of a small molecular hydrogelator derived from isophthalic acid, which contains urea as a functional group [30]. Finally, and on the basis of our previous study focused on nanoemulsions [12], the combination of vitamin E-loaded hydrogels along with vitamin E-loaded nanoemulsion were assessed in the context of assisted reproductive techniques.

## 2. Materials and Methods

### 2.1. Reagents and Media

Flow cytometry equipment, software, and consumables were purchased from Beckman Coulter (Fullerton, CA, USA). The remaining chemicals (Reagent grade or higher), vitamin E (CAS number 10191-41-0), Polaxamer 127, PI (propidium iodide), FeSO_4_ 7H_2_O, and Transwell^®^ permeable supports were obtained from the Sigma Chemical Co. (Madrid, Spain). Epikuron 145 V was purchased from Cargill (Barcelona, Spain), while the other fluorescent probes were purchased from Thermo Fisher Scientific (Barcelona, Spain). Ethanol was used to prepare a 10 mM stock solution of Trolox (vitamin E analogue). The oxidant stock solution was prepared as 10 mM FeSO4 and 50 mM sodium ascorbate (Fe^2+^ ascorbate) in water. The stock solutions of the fluorescence probes were as follows: PI, 1.5 mM in Milli-Q water; YO-PRO-1, 50 μM in DMSO; CM-H_2_DFCDA, 0.5 mM in DMSO; Mitotracker deep red, 1 mM in DMSO; C11-Bodipy 581/591, 0.2 mM in DMSO. All the fluorescent stocks were kept at −20 °C, in the dark until needed. The freezing extender was prepared in our laboratory, as described previously [10], using reagent-grade chemicals purchased from Panreac Química S.A. (Barcelona, Spain) and the Sigma Chemical Co. (St. Louis, MO, USA). The bovine gamete medium (BGM) was composed of 87 mM NaCl, 3.1 mM KCl, 2 mM CaCl_2_, 0.4 mM MgCl_2_, 0.3 mM NaH_2_PO_4_, 40 mM HEPES, 21.6 mM sodium lactate, 1 mM sodium pyruvate, 50 μg/mL kanamicine, 10 μg/mL phenol red, and 6 mg/mL BSA (pH 7.5).

Hydrogelator HG was produced following the synthetic procedures described previously [31]. Raw materials were purchased from Sigma Aldrich and used as received.

### 2.2. Vitamin E Nanoemulsion Formulation

Vitamin E nanoemulsions (NVEs) were formulated following the protocol described by Sanchez-Rubio et al. (2020) [12]. The nanoemulsions were obtained by employing controlled emulsification, when an organic phase comprised ethanol/acetone (1:19), vitamin E (58 mg) and Epikuron 145V (20 mg) were poured onto 10 mL of a 0.25 *v*/*w* aqueous solution of Poloxamer 127. The solution was subjected to magnetic stirring for 10 min, and finally, the solvents were evaporated using a rotary evaporator [21]. A Z-sizer NanoZS from Malvern (UK) was employed to obtain the following physicochemical characteristics: hydrodynamic mean diameter, polydispersity index (PDI), and Z-potential.

### 2.3. Stability Studies of the Vitamin E Nanoemulsions

For the stability studies, 100 µL of the NVEs were incubated at 37 °C in 1 mL of PBS or 1 mL of BGM, and the hydrodynamic radio (R_H_) and PDI were determined over time using dynamic light scattering (DLS) measurements.

### 2.4. Release Studies

#### 2.4.1. Vitamin E Nanoemulsions

One milliliter of NVE suspension was placed on dialysis membrane (molecular weight cut off: 3500 KDA) and incubated in 10 mL of BGM. The suspension was incubated at 37 °C with continuous stirring (50 rpm) in an IKA KS 3000 incubator shaker. Three milliliters of release medium was removed at different intervals of incubation to measure the concentration using a spectrophotometer at 275 nm.

#### 2.4.2. Vitamin E-Loaded Hydrogels

Here, 0.25 mL of vitamin E-loaded hydrogels (HVEs) and 2.75 mL of PBS (pH 7.4) were placed in a spectrophotometer cuvette and incubated at 37 °C. The amounts of vitamin E released to PBS were measured at different intervals of incubation using a spectrophotometer at 275 nm.

### 2.5. Transwell^®^ Design and Vitamin E Hydrogel Formulation

**Figure 1:**
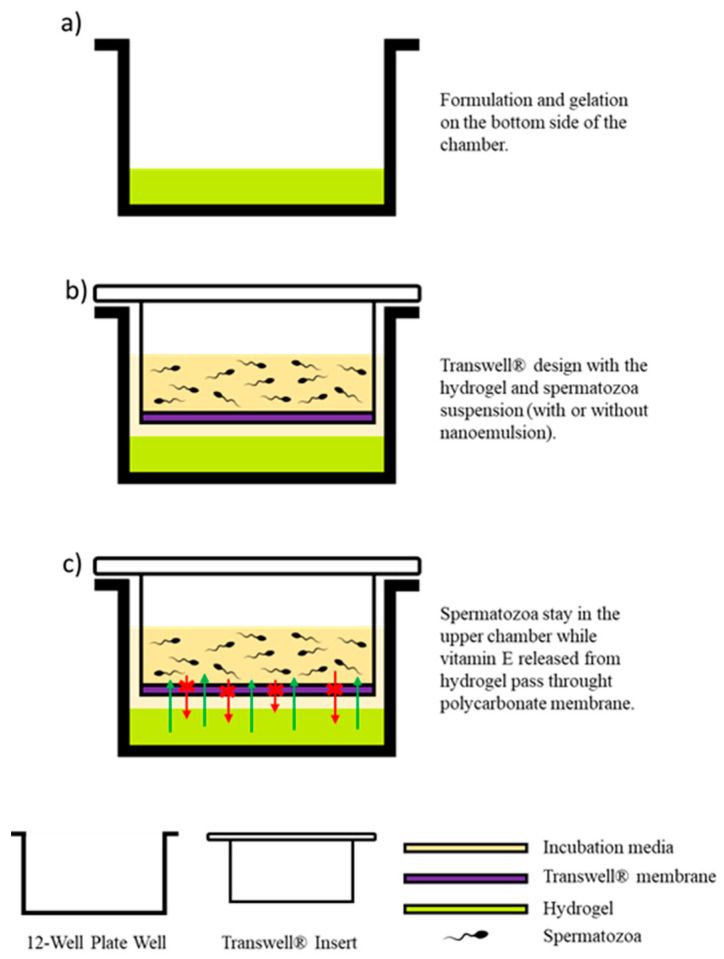
Design of the transwell system. (**a**) Gelation scheme on the plate well; (**b**) Sperm sample incubation into the transwell insert to avoid the mixture with the hydrogel; (**c**) Scheme of vitamin E and sperm dynamics across the polycarbonate membrane.

A new incubation method that utilizes a transwell chamber was developed for the incubation of sperm cells in the presence of hydrogels (see Figure 1). The need to develop this system arose from the requirement of preventing sperm from becoming embedded within the hydrogel structure. The system designed to generate vitamin E-loaded hydrogels (HVEs) was the following: Hydrogels were obtained using slow pH change generated by the well-controlled hydrolysis of glucono-δ-lactone (GDL). Then, 10 mg of HG hydrogelator were dissolved in 1 mL of 70 mM aqueous NaOH and 0.25 mg of vitamin E (Trolox) were added to the solution to attain 1 mM of free vitamin E analogue (Trolox). Next, the solution was slowly acidified by adding 2 mg of GDL [30]. The resulting solutions were poured into the bottom of the chamber and were stored for 24 h to allow complete gelation. Hydrogels were maintained at 4 °C until use. Finally, a spermatozoa suspension (with or without nanoemulsions) was placed in the upper chamber (12 mm diameter insert), over a 0.4 µm pore-size polycarbonate membrane. The entire system was embedded using BGM.

This system allows only the flow of vitamin E from the gelled hydrogel into the liquid culture through the microporous membrane, thus, avoiding contact between the spermatozoa and the hydrogel.

### 2.6. Animals, Sperm Collection, and Cryopreservation

Sperm samples were recovered from the epididymides of six mature stags (age >4.5 years, weight >130 kg) that were legally culled and hunted in their natural habitat during the rutting season (September–October) [18]; the samples were frozen following the protocol described by Fernández-Santos et al. (2007) [10]. No ethical approval was considered necessary; the data provided in the present research were attained in compliance with the Ethical Principles in Animal Research. Animal handling and protocols were performed according to the guidelines approved by the Spanish Ministry of Presidency (RD53/2013), which conforms to the European Union Regulation 2010/63.

### 2.7. Experimental Design

The frozen ejaculates from six stags were used in this experiment (200 × 10^6^ m/per straw). Two straws were thawed (37 °C for 30 s) for each male and the freezing extender was washed out by diluting it with three volumes of BGM (600× *g*, 5 min, room temperature) and removing the supernatant. The pellet was resuspended in BGM up to 30 × 10^6^ m/mL [15].

Once washed, the sample was divided equally into five tubes. One was left as a control and was analyzed immediately. The second treatment was 1 mM of free vitamin E, as a second control of the effect of free vitamin E. The subsequent treatments were 9 mM of vitamin E nanoemulsions (NVEs) and 1 mM of vitamin E hydrogels (HVEs). The last treatment was the combination of 1 mM HVEs and 9 mM NVEs (H + N) (see Figure 1). The processing of the five tubes was replicated and the oxidant solution supplementation occurred at a final concentration of 100 μM Fe^2+^ and 500 μM ascorbate. The tubes were incubated at 37 °C and analyzed for sperm motility, viability and apoptosis-like changes, mitochondrial activity, lipid peroxidation, ROS production, and DNA damage after 2, 5, and 24 h.

### 2.8. Sperm Motility Analysis

Sperm motility was measured using a Sperm Class Analyzer^®^ (SCA V6.2, Microptic S.L, Barcelona, Spain) at 37 °C. Samples were diluted to 10–20 × 10^6^ spermatozoa/mL, and a Makler counting chamber (10 μm depth) was employed. A minimum of 5 fields and 300 spermatozoa were captured per sample at 25 frames/s. The image sequences were saved and later evaluated. All software settings were adjusted to stag spermatozoa. Samples were analyzed for total (TM) and progressive (PM) motility, and the following specific kinematic parameters: VCL, velocity according to the actual path (μm/s); VSL, velocity according to the straight path (μm/s); VAP, velocity according to the smoothed path (μm/s); STR, straightness (%); ALH, amplitude of the lateral displacement of the sperm head (μm); and BCF, head beat-cross frequency (Hz).

### 2.9. Fluorescence Probes for Sperm Analysis

#### 2.9.1. Sperm Viability and Apoptosis-like Changes

Sperm viability and apoptotic-like status were analyzed following the protocol described by Martínez-Pastor et al. (2009) [32], and three subpopulations were obtained: viable (unstained, YO-PRO-1-/PI-); apoptotic-like membrane changes (YO-PRO-1+/PI-); and non-viable (membrane damaged, PI+) [33].

#### 2.9.2. Mitochondrial Activity Assessment

The mitochondrial status was assessed using a Mitotracker Deep Red 633 fluorescent probe (0.1 μM). The MT+ population was defined as the population of spermatozoa with active mitochondria [4]. Stained samples were incubated (30 min, darkness) prior to assessment.

#### 2.9.3. Lipid Peroxidation Assessment

A C11-BODIPY 581/591 fluorescent probe was used to estimate the susceptibility of sperm to lipid peroxidation, as described by Domínguez-Rebolledo et al. (2010) [34]. An aliquot of each treatment was incubated (30 min, darkness, 37 °C) with C11-BODIPY 581/591 (2 μM). The samples were washed by means of centrifugation (600× *g*, 5 min) and were extended in BGM.

#### 2.9.4. Reactive Oxygen Species Production

Reactive oxygen species production was assessed using the CM-H_2_DCFDA fluorescent probe, following the protocol described by Del Olmo et al. (2015) [33]. A sperm suspension of each treatment (1 × 10^8^ cells/mL) was incubated (37 °C, 20 min, darkness) with CM-H_2_DCFDA (0.5 mM) in BGM. The median H_2_DCFDA fluorescence of the viable sperm population value was noted.

#### 2.9.5. Sperm Chromatin Structure Assay (SCSA^®^)

A Chromatin stability assessment was carried out, as previously described by Evenson et al. (2016) [35]. This technique is based on the susceptibility of DNA to acid-induced denaturation and the metachromatic property of dye orange acridine (green, dsDNA; double strand vs. red, ssDNA; single strand).

DNA fragmentation index (DFI, % of spermatozoa with DFI >25) and high DNA stainability (HDS, % of spermatozoa with green fluorescence higher than channel 600, of 1024 channels) were obtained using flow cytometry [36].

### 2.10. Flow Cytometry Analysis

Two flow cytometers were employed. The following tests were carried out using a FlowSight^®^ imaging flow cytometer (Amnis, Merck-Millipore, Germany) controlled with the INSPIRE^®^ software (v.3): sperm viability and apoptosis-like changes, mitochondrial activity, lipid peroxidation, and ROS production. An FC-500 (Beckman Coulter, Brea, CA, USA) controlled with MXP software (v.3) was used to analyze SCSA^®^. IDEAS^®^ software and WEASEL software (WEHI, Melbourne, Australia) were used to analyze the raw data. Ten thousand events were acquired per sample and the lasers used to excite the fluorochromes were: a 633 nm helium-neon laser for Mitotracker Deep Red, a 405 nm violet laser for Hoechst 33,342 and a 488 nm laser for the remaining fluorochromes. Dot plots with forward-scatter light and side-scatter light or aspect ratio and area were employed in the respective cytometers in order to exclude debris from the sperm population.

### 2.11. Statistical Analysis

The data were analyzed using an R statistical package (http://www.r-project.org (accessed on 3 November 2021)). Variables which did not have a normal distribution were transformed by means of arc sine (proportions) or were log-transformed (other variables). The effects of time, treatment (NVEs, HVEs, or H + N and/or oxidant (fixed factors) on sperm variables were analyzed using a linear mixed effects model. The model included the random effect on the male. Unless otherwise stated, the results are presented as mean ± SEM, and statistical significance was accepted for *p* < 0.05.

## 3. Results

### 3.1. Formulation and Characterization of the VE-Loaded Devices

**Scheme 1:**
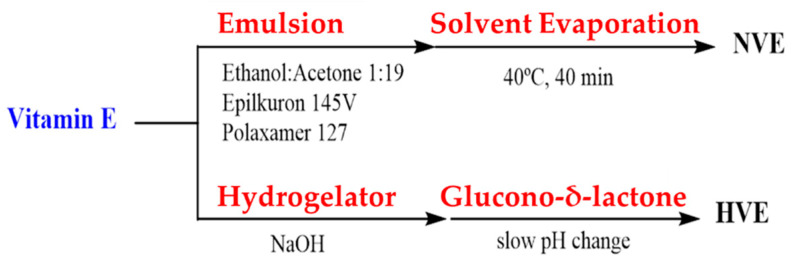
Nanoemulsion and hydrogel-based vitamin E carrier composition and release mechanism.

The NVEs were obtained following a non-expensive procedure based on mild conditions, and HVEs were obtained using slow pH change generated by the well-controlled hydrolysis of glucono-δ-lactone follow hydrogelatoring the experimental procedures reported (Scheme 1).

**Figure 2:**
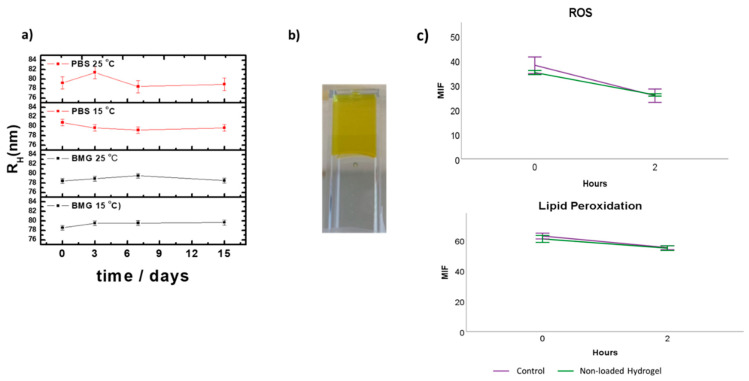
Stability and toxicity of the carriers:(**a**) Storage stability of NVEs in PBS and BGM. Data are stated as mean ± SEM from at least three independent experiments; (**b**) image of the HVEs using test tube inversion method; (**c**) differences between control and unloaded hydrogel; (**c**) vitamin E formulation effects on ROS production and lipid peroxidation. The results show MIF (mean intensity fluorescence). Plots represent the interaction of different treatments × incubation time for the flow cytometry analysis of reactive oxygen species (median fluorescence of H2DCFDA in PI− sperm) and lipid peroxidation (median green fluorescence of BODIPY C11).

The NVEs were characterized by DLS, and had an average particle size of 156 ± 1.3 nm for formulations and a very low polydispersity index (PDI) ranging from 0.117 to 0.145. The NVEs were negatively charged with a value of −18.2 ± 4.4 mV, and had high physical colloidal stability. The values of hydrodynamic radio (R_H_) and PDI of the NVEs were monitored using DLS in a 7 day experiment. The negligible increase in each parameter denoted the high stability of the formulations (Figure 2a). Furthermore, the formulation of the hydrogel systems was confirmed by the observation of no gravitational flow when employing the test tube inversion method (Figure 2b). The hydrogel had no antioxidant activity. Neither free radical production nor lipid peroxidation were different from the control (Figure 2c).

**Figure 3:**
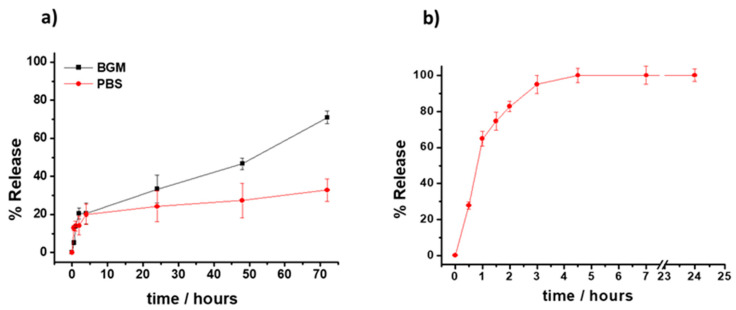
In vitro release profiles: (**a**) NVEs in PBS and BGM at 37 °C; (**b**) HVEs in PBS at 37 °C.

As shown in Figure 3, there were different patterns for the vitamin E released for each formulation. For NVEs, a significant burst release was observed during the first 5 h followed by a slow release which did not exceed 30% in PBS and 80% in BGM after 70 h (Figure 3a). In contrast, HVEs had a two-stage release mechanism, i.e., an initial release of vitamin E molecules located on the surface followed by a vitamin E release from hydrogel aggregates. This release was rapid, with a fully delivery after 3 h (Figure 3b).

### 3.2. Vitamin E Formulation Effects on Sperm Motility Assessed Using CASA^®^

**Figure 4:**
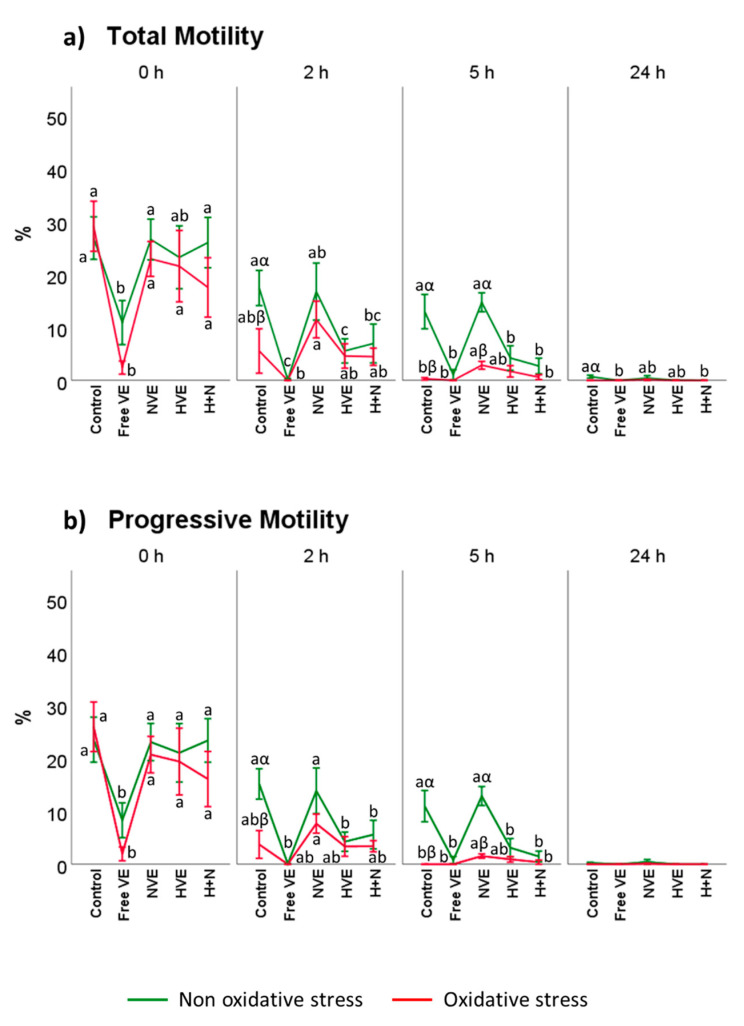
Vitamin E formulation effects on total (**a**) and progressive sperm motility (**b**). Plots represent the triple interaction of different vitamin E treatments × oxidative treatment × incubation time for CASA-derived variables. The various lowercase Latin letters (*p* < 0.05) indicate significant differences between vitamin E treatments, while the different Greek letters (*p* < 0.05) compare each treatment at the same time with a different oxidative stress status.

The overall kinematic sperm parameters were affected by supplementation (Figure 4). The controlled release devices had a stimulatory effect with respect to free vitamin E, with or without oxidative stress.

After supplementation with free VE, NVEs, HVEs, and the combination of HVEs and NVEs (H + N), total motility down to 24 h incubations in which all the samples were practically motionless (control; 0 h, 26.9 ± 3.3; 2 h, 17.5 ± 2.7; 5 h, 12.9 ± 2.2; and 24 h, 0.69 ± 0.7) *p* < 0.05) (Figure 4a). Whereas, without exogenous oxidative treatment, free VE treatment rendered the sperm practically motionless after 2 h (0.07 ± 1.5). In this sense, NVE preserved motility at similar values to the control (control 2 h, 17.5 ± 2.7; and NVEs 2 h, 16.8 ± 4.4), while HVEs (5.6 ± 5.6) and H + N (6.9 ± 2.7) had lower values as compared with the control but higher as compared with free vitamin E. Similar results were obtained after 5 h incubation at 37 °C.

When applying an exogenous source of oxidative stress, a very similar profile to that described for samples without oxidative stress was observed.

Similar results were obtained for progressive motility without applying an exogenous source of oxidation (Figure 4b). When samples were exposed to free vitamin E, progressivity was completely lost (free vitamin E 2 h, 0.8 ± 1.1 and free vitamin E 5 h, 0.7 ± 1.0). Of note, after 2 h and applying an exogenous source of oxidation, NVEs improved progressivity with significant differences from the control (*p* = 0.02). Progressivity practically disappeared after 5 h of incubation. The same effects were observed after 24 h incubation, with or without an exogenous source of oxidation.

**Figure 5:**
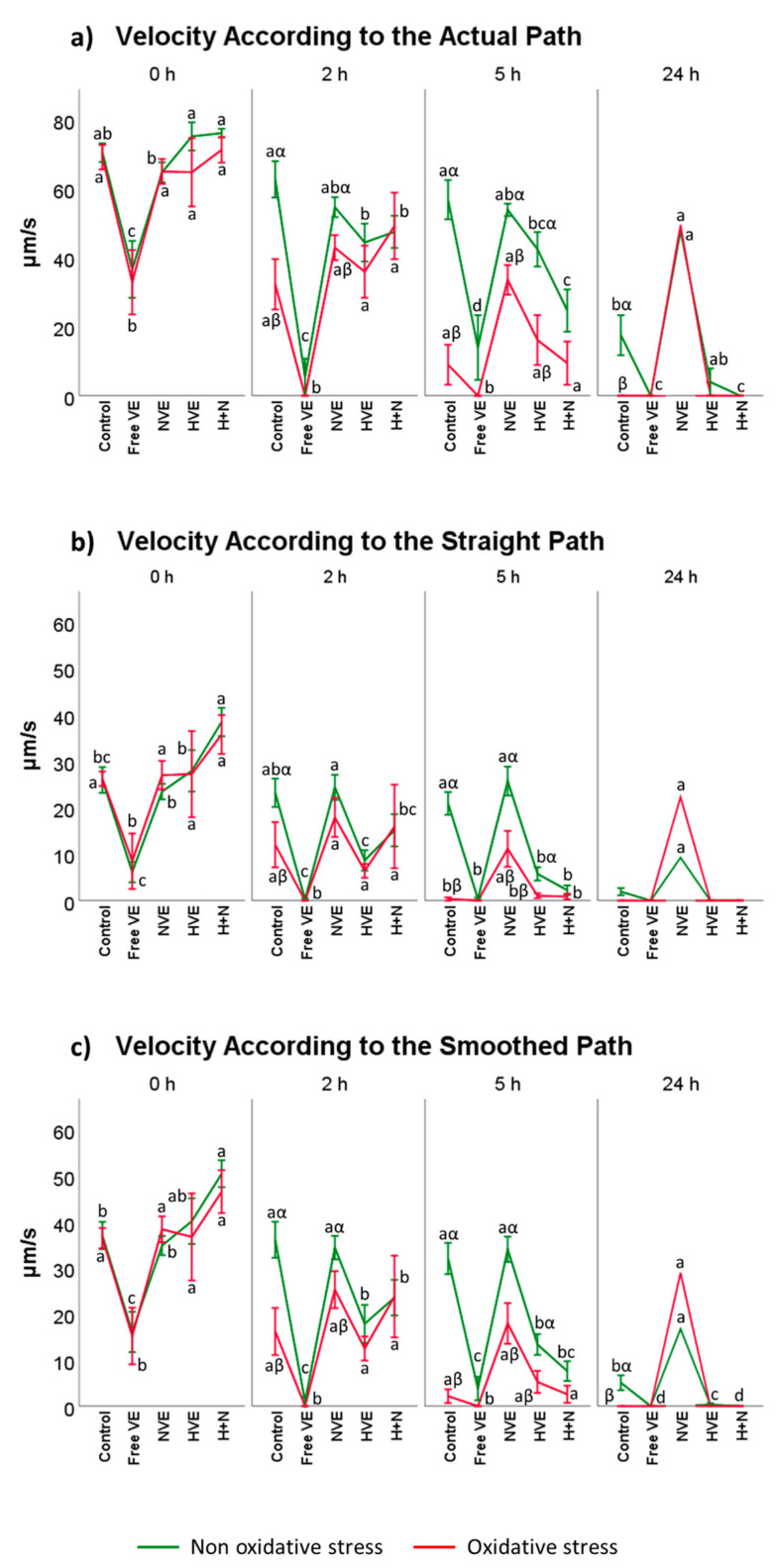
Vitamin E formulation effects on sperm velocity parameters. Plots (**a**–**c**) represent the triple interaction of different vitamin E treatments × oxidative treatment × incubation time for CASA-derived variables. The various lowercase Latin letters (*p* < 0.05) indicate significant differences between vitamin E treatments, while the different Greek letters (*p* < 0.05) compare each treatment at the same time with a different oxidative stress status.

Velocity according to the actual path (VCL), velocity according to the straight path (VSL) and velocity according to the smoothed path (VAP) (Figure 5a–c) were preserved at similar values to the control, regardless of supplementation after 2 h and 5 h incubation times following the pattern: control ≈ NVEs > H + N > HVEs. However, when applying the exogenous oxidation source, the velocity parameters improved as regards the control after 2 and 5 h of incubation following the pattern: control > NVEs > H + N > HVEs, and all treatments differed significantly regarding free vitamin E (*p* < 0.01); only NVEs maintained velocity after 24 h of incubation.

**Figure 6:**
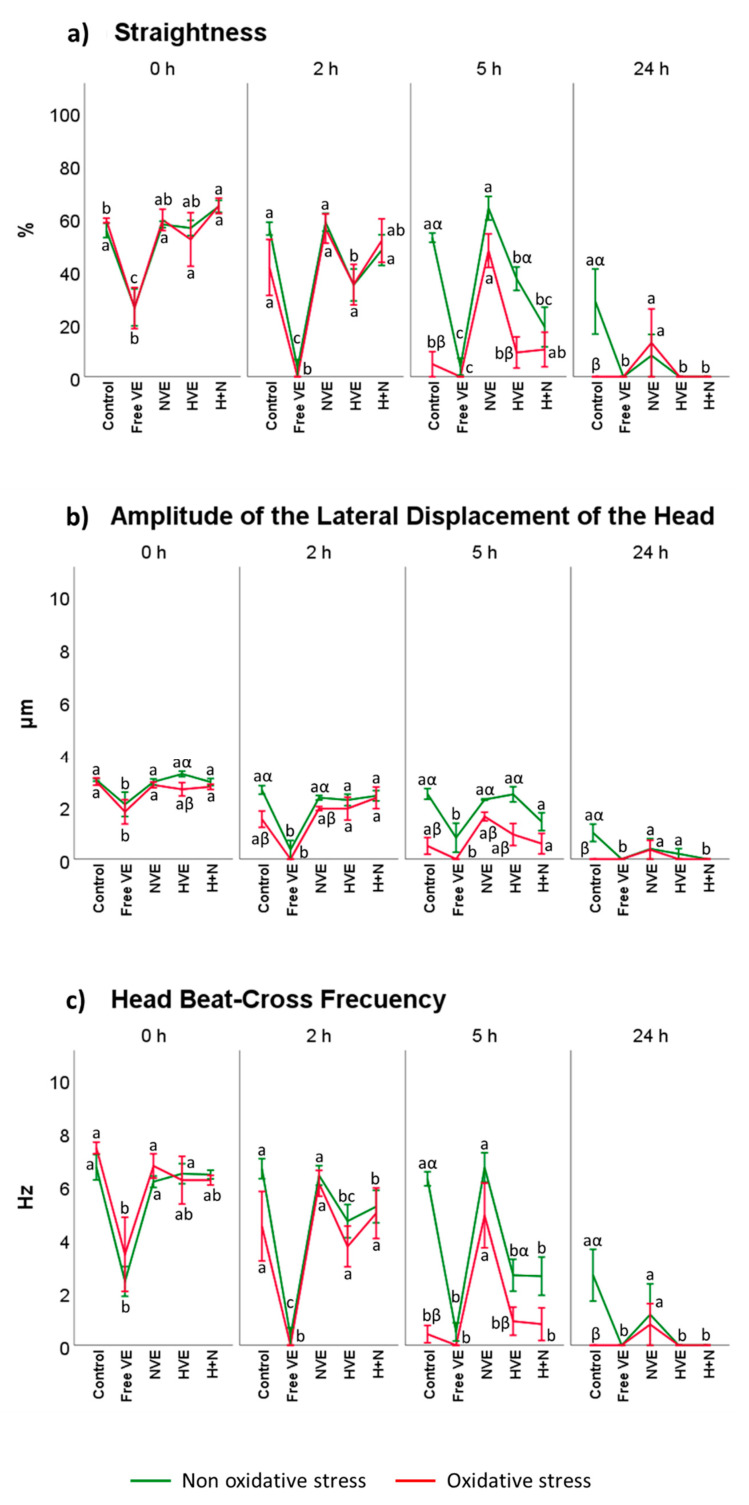
Vitamin E formulation effects on sperm kinematic parameters. Plots (**a**–**c**) represent the triple interaction of different vitamin E treatments × oxidative treatment × incubation time for CASA-derived variables. The various lowercase Latin letters (*p* < 0.05) indicate significant differences between vitamin E treatments, while the different Greek letters (*p* < 0.05) compare each treatment at the same time with a different oxidative stress status.

The linearity parameters, including the straightness variable (STR) (Figure 6a), followed the pattern: NVEs ≈ control > H + N > HVEs after 2 h of incubation with significant differences between free vitamin E and all formulation treatments (*p* < 0.02). After 5 h of incubation, the pattern changed to NVEs > control > HVEs > H + N, with significant differences between the control and HVEs (*p* = 0.004) and H + N (*p* < 0.001) treatments. Moreover, after 24 h, the linearity parameters of NVEs improved as compared with free vitamin E, with significant differences (*p* = 0.00). Upon applying an exogenous oxidation agent, after 2 h of incubation, the pattern was: NVEs > H + N > control > HVEs, and after 5 h, the pattern was: NVEs > H + N > HVEs > control, with significant differences between NVE and the other treatments (*p* < 0.05). However, after 24 h of incubation with an oxidative stress source, only NVEs maintained and greatly improved the linearity, with significant differences to free vitamin E (*p* = 0.001).

With regard to the amplitude of the lateral displacement of the sperm head (ALH) (Figure 6b), there were no significant differences among the control, NVEs, HVEs, and H + N at either 0, 2, and 5 h of incubation or with or without an exogenous source of oxidation. Finally, regarding sperm beat frequency (BCF) (Figure 6c) and after 2 and 5 h of incubation, significant differences were observed between the control vs. HVEs, and H + N (*p* < 0.01), and free vitamin E vs. all treatments (*p* < 0.02). No significant differences were found after 24 h of incubation. When applying an oxidizing agent, BCF and NVEs had the highest values, with significant differences to the other treatments at 5 and 24 h (*p* < 0.01).

### 3.3. Vitamin E Formulation Effects on Sperm Physiology

#### 3.3.1. Sperm Viability

**Figure 7:**
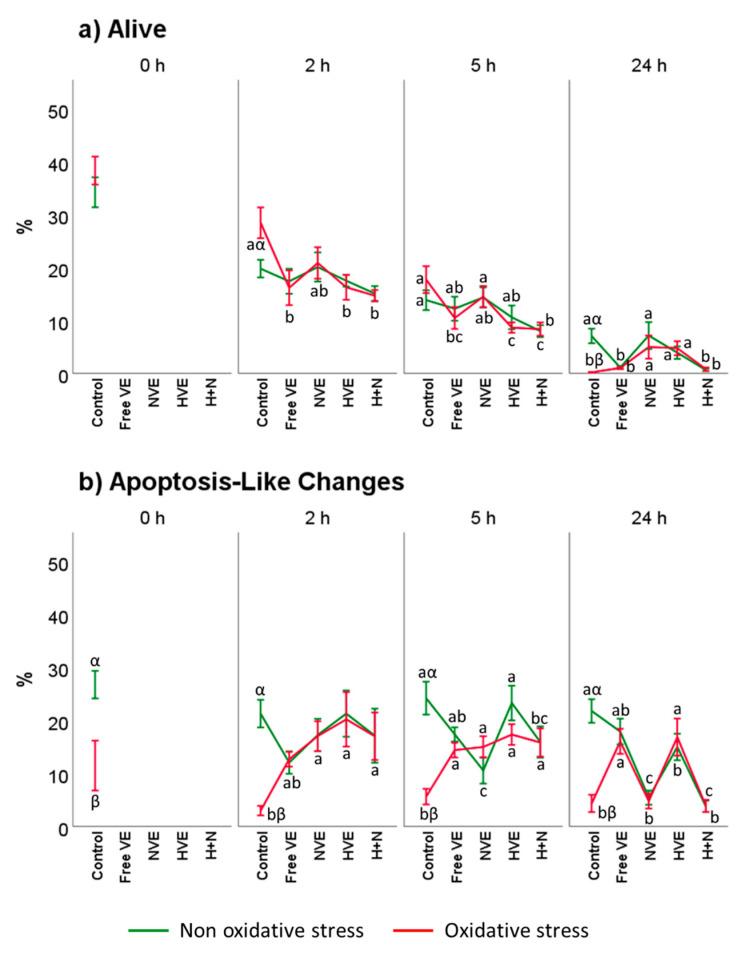
Vitamin E formulation effects on sperm viability. Plots (**a**,**b**) represent the triple interaction of different vitamin E treatments × oxidative treatment × incubation time for the flow cytometry analysis of sperm viability. The various lowercase Latin letters (*p* < 0.05) indicate significant differences between vitamin E treatments, while the different Greek letters (*p* < 0.05) compare each treatment at the same time with a different oxidative stress status.

With regard to sperm viability (Figure 7), the NVE supplementation increased the viability regarding free vitamin E at any incubation time, with and without oxidative stress. After 24 h of incubation, viability decreased drastically (control 24 h, 9.0 ± 1.1; NVEs 24 h, 9.1 ± 1.9; and HVEs 24 h, 5.9 ± 1.5) with no significant differences among them. However, H + N (0.6 ± 1.1) and free vitamin E (1.1 ± 1.2) were significantly different from the other treatments. When an exogenous oxidative agent was subjected to the spermatozoa, viability decreased proportionally in all treatments at incubation time. After 24 h of incubation, the NVE (4.9 ± 1.7) and the HVE (4.7 ± 2.0) treatments significantly maintained (*p* < 0.001) viability as compared with the control (0.2 ± 1.0) (Figure 4a). The population of apoptotic cells figure shows that (Figure 7b), the percentage increased as the incubation time progressed, with the control maintained similar values to the HVE and the H + N treatments, while the NVE treatment was meant to be the best treatment with significant differences vs. the control and vs. free vitamin E (*p* < 0.001). The same effects were observed after applying an exogenous oxidative treatment, with the NVE treatment being the only one to produce the half percentage of apoptotic cells, which was also statistically significantly (*p* < 0.001).

#### 3.3.2. Mitochondrial Activity Assessment

**Figure 8:**
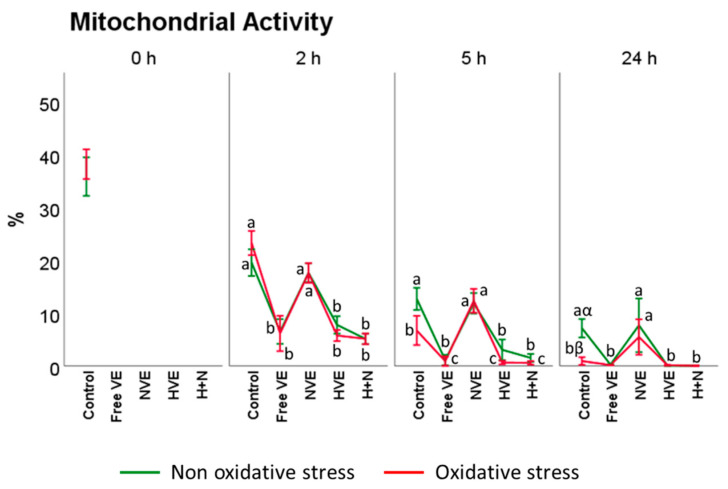
Vitamin E formulation effects on mitochondrial activity. Plots represent the triple interaction of different vitamin E treatments × oxidative treatment × incubation time for the flow cytometry analysis of mitochondrial activity (YOPRO1−/Mitotracker deep red+). The various lowercase Latin letters (*p* < 0.05) indicate significant differences between vitamin E treatments, while the different Greek letters (*p* < 0.05) compare each treatment at the same time with a different oxidative stress status.

The NVE supplementation maintained the mitochondrial activity at every incubation time at similar values to the control, while the other treatments proportionally decreased following the pattern: NVEs ≈ control > HVEs > free vitamin E > H + N, with significant differences among the NVE and HVE, free vitamin E, and H + N treatments (*p* < 0.01). When an exogenous oxidative source was applied, the NVE treatment had greater mitochondrial activity than the control following the pattern: NVEs > control > free vitamin E > HVEs > H + N, where the NVE treatment and the control had significantly greater mitochondrial activity than the other treatments (*p* < 0.01) (Figure 8).

### 3.4. Vitamin E Formulation Effects on Lipid Peroxidation, Intracellular ROS, and DNA Damage of Thawed Spermatozoa

**Figure 9:**
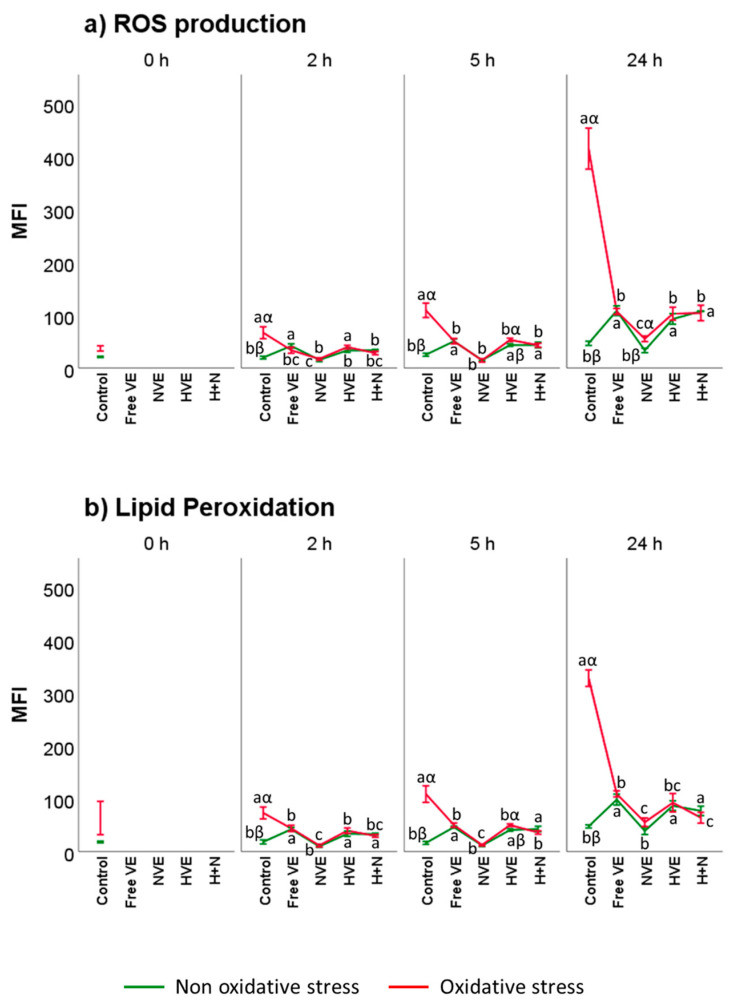
Vitamin E formulation effects on ROS production (**a**) and lipid peroxidation (**b**). The results show MFI (mean fluorescence intensity). Plots represent the triple interaction of different vitamin E treatments × oxidative treatment × incubation time for the flow cytometry analysis of reactive oxygen species (median fluorescence of H2DCFDA in PI− sperm) and lipid peroxidation (median green fluorescence of BODIPY C11). The various lowercase Latin letters (*p* < 0.05) indicate significant differences between vitamin E treatments, while the different Greek letters (*p* < 0.05) compare each treatment at the same time with a different oxidative stress status.

Intracellular ROS production (Figure 9a), which was measured as the median H_2_DCFDA fluorescence of the viable sperm population, increased at every incubation time in the medium under oxidative stress conditions, except in samples supplemented with the NVE treatment, which significantly decreased ROS levels (*p* < 0.001) (Figure 9a). Furthermore, the free vitamin E without oxidative stress significantly increased ROS levels despite other treatments (*p* < 0.001), following the pattern: free vitamin E > HVEs ≈ H + N > control > NVEs. After subjecting samples to an exogenous oxidative agent, the percentage of ROS increased dramatically in the control, especially at the highest incubation time, showing that all the VE formulations had efficacy as powerful antioxidants, with the NVE treatment standing out, following the pattern: control > free vitamin E > HVEs ≈ H + N > NVEs, with significant differences between the control and the other treatments (*p* < 0.001), and the NVEs and the other treatments (*p* < 0.001).

These ROS measurements were reflected in a similar profile for lipid peroxidation (LPO) (Figure 9b). As described above, values increased at every incubation time in the medium, with the NVEs being the best antioxidant treatment, following the same pattern: free vitamin E > HVEs > H + N > control > NVEs, and with significant differences between NVEs and the other treatments (*p* < 0.001). With the oxidative treatment, all the VE formulations also showed their high efficacy as regards preventing lipid peroxidation, following the pattern: control > free vitamin E > HVEs > H + N > NVEs, with significant differences between these treatments and the control at any incubation time (*p* < 0.001).

**Figure 10:**
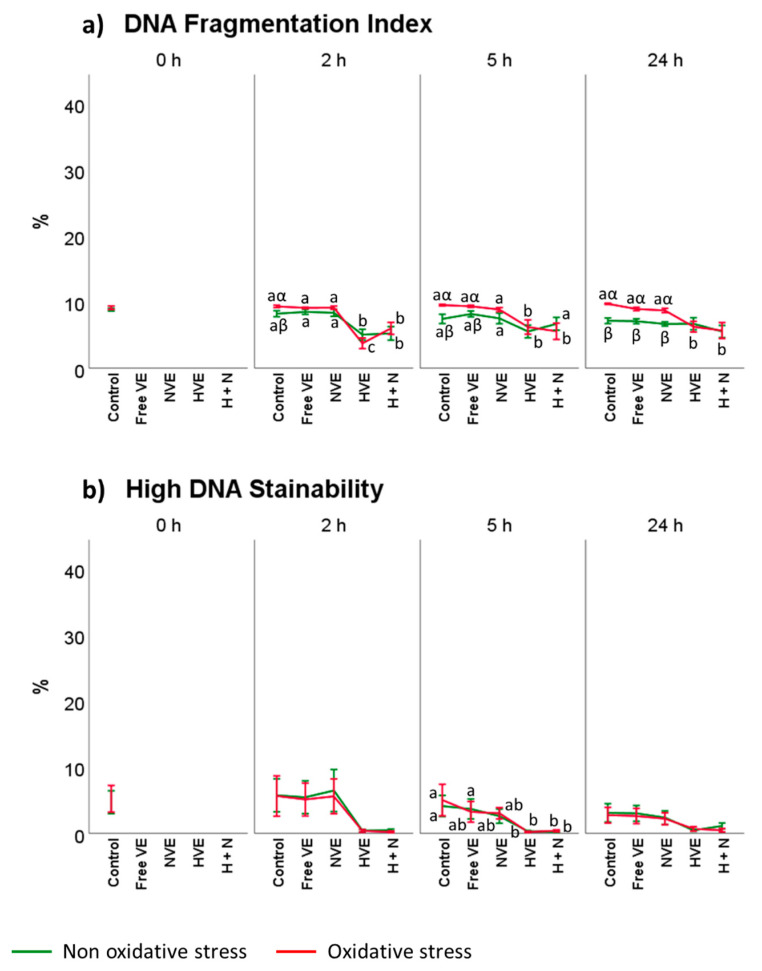
Vitamin E formulation effects on DNA. Plots (**a**,**b**) represent the triple interaction of different vitamin E treatments × oxidative treatment × incubation time for the flow cytometry analysis of DNA fragmentation index and high DNA stainability (median fluorescence of sperm assessed by SCSA^®^). The various lowercase Latin letters (*p* < 0.05) indicate significant differences between vitamin E treatments, while the different Greek letters (*p* < 0.05) compare each treatment at the same time with a different oxidative stress status.

DNA fragmentation was measured using SCSA^®^. The DFI values were all very similar for every treatment at any incubation time, as shown in Figure 10a.

## 4. Discussion

The results from this study support our hypothesis that the antioxidant capacity of vitamin E-loaded hydrogels, nanoemulsions, and a combination of both are able to neutralize ROS production and lipid peroxidation under oxidative stress conditions and to improve sperm motility with respect to free vitamin E, with and without oxidative stress. The latter is a recurrent issue to address, because hydrophobic molecules require the use of organic solvents which are, in turn, toxic for the spermatozoa [7,8,9,10,11,12,37,38,39].

An imbalance between ROS production and antioxidant capacity is critical for sperm cells; the low antioxidant level may have no effect, and high concentrations may inhibit the beneficial physiological effects of ROS [40,41]. However, insufficient protection against ROS leads to lipid peroxidation [42], which is clearly reflected by the loss of motility [43], mitochondrial membrane damage [44], DNA damage [45], and entering the intrinsic apoptotic cascade [46].

In this scenario, nanotechnology can help develop safer and more efficient treatments, avoiding the limitations of traditional antioxidants. Vitamin E drug delivery systems have beneficial effects on kinetic parameters, while treatment with free vitamin E is deleterious to semen, possibly owing to the use of organic solvents, as has been demonstrated previously with guineas pigs (*Cavia porcellus*) [37], male albino rats (*Sprague dawley*) [38], red snappers (*Lutjanus argentimaculatus*) [39], and red deer (*Cervus elaphus hispanicus*) spermatozoa [7,8,9,10,11]. The use of HVEs and H + N also improves the results obtained with free vitamin E, which is a critical advance, since motility is essential for fertilization, during which changes and maintenance as regards the linear and progressive motility of sperm are crucial [41,47]. The results are not great as compared with NVEs. In the case of HVEs, this could be explained by the fact that the low vitamin E loading chosen for the generation of the hydrogels was not sufficiently high. Moreover, the results using H + N could be explained on the basis of an interaction between nanoemulsions and hydrogel.

The results obtained for ROS production and lipid peroxidation are consistent with those related to mitochondrial activity, viability, and DNA damage. In this respect, free vitamin E significantly reduced mitochondrial activity regarding the control and NVEs, at 2, 5, and 24 h, while NVEs provide a better protection against ROS production and lipid peroxidation. When subjected to exogenous oxidative treatment, NVEs are still the treatment that best maintains mitochondrial activity, as reported in our previous study [12]. Our results are consistent, in that, an increase in mitochondrial activity is also observed in response to an improvement in motility, since in order for the spermatozoa to carry out a correct motility they need a synergy with the mitochondrial one, requiring an effective ATP production. Our results show a first approximation of the benefits of nanotechnology for mitochondrial activity, since it, and thus motility, are maintained because a functional mitochondrion will provide the spermatozoon with sufficient energy to carry out the motility that is so necessary for sperm function. The results obtained after employing hydrogels show that this approach can also maintain the beneficial effects of free vitamin E. Among other aspects, it protects the active molecule, resulting in more effective therapies. The effectiveness of nanotechnology on reproduction has also been reported by other research groups. Human spermatozoa are one of the most frequently studied, with equally promising results to those obtained in this study. Ferreira et al. (2018) recently reported micelles comprising glycerophospholipid mixtures as an approach to reduce oxidative stress [48], while zinc oxide (ZnO) nanoparticles have antioxidant effects in human semen without affecting either sperm chromatin or motility [49]. A recent study carried out with cerium oxide (CeO_2_) nanoparticles in sheep (*Ovis aries*) spermatozoa reported beneficial effects on kinematic and morphological parameters with no cytotoxic effects on sperm, both in the short term [50] and up to a 96 h incubation period [51], but the protection cannot be related to an antioxidant activity of the CeO_2_ nanoparticles, since the ROS levels in the exposed cells were similar to those of the unexposed cells [51]. In our study, the results obtained for DNA fragmentation and stainability were no different to those obtained in other studies with different antioxidants, such as free vitamin E [7,8,9,10], melatonin [8,9], and cinnamtannin B-1 [17].

One issue intensely researched in nanotechnology is safety. The safety of a particular nanocarrier is determined by the characteristics of the nanomaterial and by the biological system. Better knowledge of the effects of these nano-systems in reproduction is necessary, owing to the very different outcomes reported. In the present study, the use of hydrogels and nanoemulsions did not have a detrimental effect on sperm physiology, corroborating the positive effects shown in our previous study with vitamin E nanoemulsions [12]. These non-harmful/beneficial effects have been repeatedly demonstrated in independent studies. Zinc oxide nanoparticles (ZnONPs) have been shown to not penetrate the membrane of spermatozoa and were beneficial to spermatozoa as they withstood the freeze-thaw process better than samples without supplementation, without any adverse effects in human samples [49]. Other groups have recently tested the effects of cerium oxide nanoparticles (CeO_2_NPs) on ram (*Ovis aries*) spermatozoa, with protective effects on sperm motility after 48 h and up to 96 h of incubation [50,51]. Moreover, chitosan-dextran sulphate nanoparticles (CS-DSNPs) for GnRH release were tested in a rabbit (*Oryctolagus cuniculus*) insemination extender. These NPs did not affect rabbit semen motility and the acrosome integrity was improved [52]. Similarly, de Castro Jorge Silva et al. (2017) demonstrated that chitosan-coated lipid-core nanocapsules (LNC-CS) did not have a toxic effect on ram (*Ovis aries*) spermatozoa [53], while Ferreira et al. (2018) showed that the incubation of human sperm with micelles made from glycerophospholipid mixture increased sperm motility and resistance to oxidative stress [48]. However, the nanocarriers used in cosmetics, electronics, and food packaging have had reprotoxic effects [54], with harmful effects on sperm function. Xu et al. (2014) discovered that exposure to silica nanoparticles (SNPs) caused damage to the maturation process of mouse (*Mus musculus*) spermatozoa [55]. Talebi et al. (2013) warned that the employment of zinc oxide nanoparticles (ZNPs) acted as testicular toxicant on mouse (*Mus musculus*) spermatogenesis [56], while Hong et al. (2015) published that the usage of titanium oxide (TiO_2_NPs) may be associated with alterations in testicular marked enzyme and oxidative stress in mice [57]. These studies explain that different types of nanocarriers have different impacts on sperm functions according to the chemical nature of the nanomaterial and the biological target. Better knowledge of the effects of nanotechnology on fertility in both humans and animals is, therefore, crucial. Nevertheless, the results presented in our studies, together with those carried out by other research groups, show that nanotechnology, when applied in a logical manner, is a safe tool that can help improve sperm quality. Our results show that hydrogels are an interesting and safe alternative to traditional antioxidants.

## 5. Conclusions

The addition of VE to sperm cells prevents oxidative stress in sperm cells but renders spermatozoa practically motionless. In this regard, our study shows, for the first time, that VE-loaded hydrogels, nanoemulsions, and a combination of both, could be an advantageous strategy against oxidative damage in spermatology as compared with the addition of free VE. The beneficial effects of these devices include improvement to sperm motility parameters with respect to free VE, and a reduction in ROS production and lipid peroxidation with respect to a control under oxidative stress conditions. These results inspire us to consider the usage of antioxidants by combining different nanostructures and opens up new horizons with regards to the applications of nanotechnology in reproduction.

## Data Availability

The data presented in this study are available in article.
